# WHEN POLICY INTENTIONS GO ASTRAY: THE DEPLOYMENT OF HOME CARE AND HOME SUPPORT MARKET TOOLS IN FRANCE AND QUEBEC

**DOI:** 10.1093/geroni/igad104.2863

**Published:** 2023-12-21

**Authors:** Patrik Marier, Loïc Trabut

**Affiliations:** Concordia University, Montreal, Quebec, Canada; INED, Paris, Ile-de-France, France

## Abstract

Using Gingrich’s (2011) typology of welfare markets, this contribution studies the selection and enactment of market tools in two different jurisdictions (France and Quebec). Quebec represents a classic example of a managed market with regional health authorities (CISSS/CIUSSS) seeking the lower the cost of long-term care by contracting services to community groups and the private sector. These contractual agents have gradually replaced health and social care professionals within the public system. France has opted to enact a consumer driven market with the introduction of the allocation personnalisée d’autonomie (APA) which provides cash benefits to eligible older adults who can then select the provider of their choice. Both of these market reforms are compared and analyzed across three types of territories : rural (Finistère and Bas-St-Laurent), urban (Paris and Montreal), and in industrial decline (Somme and Mauricie). Despite operating within highly differentiated long-term care policy frameworks and market tools, both jurisdictions face similar difficulties in the enactment of market mechanisms. For instance, rural territories fail to generate sufficient providers to develop a market where a regional health authority (Quebec) or older adults (France) can actually select a provider and negotiate terms of service. Public authorities must then intervene to generate market-like conditions. Regardless of the jurisdiction, the conceptualization of market mechanism has clearly an urban setting in mind making them ill-suited for other environment and there is gradual movement towards the development of austerity market where individuals are forced to seek alternatives beyond the market tools deployed by governments.

